# Exploring the neural mechanisms of electroacupuncture for cognitive impairment in depression using functional near-infrared spectroscopy: a randomized controlled trial

**DOI:** 10.3389/fpsyt.2025.1650695

**Published:** 2025-09-24

**Authors:** Jie Yuan, Rongrong Qi, Yuting Zhang, Xinghua Ma, Tian Zhao, Ying Sun, Tingting Yang, Yaling Lei

**Affiliations:** ^1^ Shaanxi Provincial Hospital of Traditional Chinese Medicine, Xi’an, China; ^2^ Shaanxi University of Chinese Medicine, Xianyang, China

**Keywords:** depression, electroacupuncture, cognitive impairment, PDQ-D, fNIRS, RCT

## Abstract

**Background:**

Depression is a prevalent psychiatric disorder that is commonly associated with a high risk of recurrence and suicide. One of its core symptoms is cognitive impairment, which can occur during the prodromal, acute (76.9 – 94.0%), and remission phases (32.4 – 44.0%). This impairment substantially contributes to both health and socioeconomic burdens. Recent evidence suggests the therapeutic potential of electroacupuncture; however, its adjunctive efficacy compared with that of standard pharmacotherapy remains ambiguous. This study aimed to assess the effectiveness and safety of using conventional antidepressants alone versus in combination with electroacupuncture.

**Methods:**

This multicenter, prospective, open-label randomized controlled trial enrolled 120 patients diagnosed with depression. The patients were randomly assigned at a 1:1 ratio to receive either conventional pharmacotherapy alone or pharmacotherapy combined with electroacupuncture. The intervention lasted for four weeks, followed by a post-treatment observation period. The Perceived Deficits Questionnaire for Depression (PDQ-D) was the primary outcome measured. The 17-item Hamilton Depression Rating Scale (HAMD-17), N-back task, Stroop Color-Word Test (SCWT), Trail Making Test-Part B (TMT-B), and functional near-infrared spectroscopy (fNIRS) indicators, such as the concentration of oxyhemoglobin (oxy-Hb), integral values, and centroid values, were the secondary outcomes assessed. R 4.5.0 was used to conduct the analyses.

**Results:**

A total of 103 of the 120 participants completed the study. Significant main effects of time and time ×group interactions across most outcomes (P < 0.05) were revealed using linear mixed-effects modeling. The electroacupuncture group demonstrated significantly lower PDQ-D scores (P < 0.05) post-treatment, indicating greater cognitive improvement. This group also demonstrated superior performance in HAMD-17, N-back, SCWT, TMT-B, and fNIRS metrics. Most group main effects were non-significant (P > 0.05); however, the interaction effects indicated a stronger response to the combined intervention.

**Conclusion:**

Compared with pharmacotherapy alone, the combination of pharmacotherapy with electroacupuncture improved cognitive symptoms, mood, and executive function more effectively in patients with depression over a short period. In addition, this combination was associated with enhanced cortical activation on fNIRS. The combined treatment was found to be safe and effective, suggesting promising implications for clinical practice and future research.

**Clinical trial registration:**

https://www.chictr.org.cn/hvshowproject.html?id=250167&v=1.0, identifier ChiCTR2400082987

## 1 Introduction

Psychological disorders—including anxiety, depression, and stress-related pathologies. According to the Global Burden of Disease Study 2021, mental disorders account for about 155.4 million disability-adjusted life years (DALYs) globally (1). These disorders are influenced by a complex interplay of genetic susceptibility, hormonal imbalance, neurocircuitry dysregulation (particularly in prefrontal-hippocampal pathways)and environmental stressors (2). Among psychological disorders, depression is particularly prevalent and poses significant public health and economic burdens globally. Its etiology remains multifactorial and involves life stress, chronic diseases (e.g., hypertension, diabetes), pain, drug reactions, and genetic vulnerabilities (3). Clinically, depression is associated with diverse affective, somatic, and cognitive symptoms (4, 5). Notably, cognitive impairment—particularly in executive function, attention, memory, and processing speed—is a core feature of depression. Evidence indicates that dysfunction in the prefrontal cortex (PFC), the central hub for emotional and cognitive regulation, directly contributes to the persistence of these deficits during remission (6) (7, 8). Therefore, regular cognitive function assessment and targeted cognitive interventions are essential.

Pharmacological treatments, especially selective serotonin reuptake inhibitors, have currently shown some efficacy in alleviating depressive symptoms. Among these, because of its rapid onset, stable efficacy, and favorable safety profile, paroxetine hydrochloride is widely used clinically (9). However, pharmacotherapy alone has certain limitations, such as delays in onset, adverse effects, interindividual variability, suboptimal responses in some patients, high costs, and long-term dependence risk. This underscores the need for safer, more effective, and individualized strategies of treatment (10, 11).

Acupuncture, a traditional Chinese therapy with a long history, has achieved modern clinical recognition for its safety and adjunctive efficacy (12, 13). Electroacupuncture (EA), which integrates traditional needle insertion with low-frequency electrical stimulation, has gained increasing attention in recent years for the treatment of depression because of its increased intensity of stimulation and consistency at acupoints. This modality has greater potential for neuromodulation than manual acupuncture does (14, 15). Emerging evidence has shown that EA exerts antidepressant effects and rapidly alleviates core depressive symptoms (16). However, treatment protocol standardization remains inadequate, as there is no consensus on parameters such as the frequency of stimulation, duration of therapy, or intervention intensity. This lack of uniformity hinders the objective evaluation of efficacy and limits broader clinical adoption. Additionally, direct comparative studies between the use of antidepressants alone and combined therapy with EA and pharmacological treatment remain scarce, making it challenging to analyze the best therapeutic approach. Thus, to further validate and refine the application of acupuncture in depression, well-designed and rigorously controlled clinical studies are urgently needed.

The rapid evolution of the Internet of Things (IoT) in smart healthcare has established non-invasive wearable sensors as pivotal tools for disease monitoring (17).Widely adopted devices—including electrocardiogram (ECG) monitoring bands and smart belts—now enable real-time tracking of both physical and mental health parameters, offering efficient solutions for continuous physiological surveillance (18, 19).Within this technological landscape, functional near-infrared spectroscopy (fNIRS) has emerged as a transformative wearable neuroimaging modality capable of objectively evaluating cortical activity in depression (20). This non-invasive, portable technique quantifies task-evoked changes in cerebral oxygenation(oxy-Hb), providing real-time insight into brain function. Critical parameters—such as oxy-Hb concentration, integral value (reflecting activation intensity), and centroid value (indicating temporal dynamics)—deliver physiological insights that complement and surpass conventional subjective scales and behavioral tasks (21). These objective metrics align with medical intelligence frameworks that decode depression’s complexity through multimodal biomarkers. By facilitating dynamic, task-based assessment of prefrontal activation, fNIRS significantly enhances precision in tracking therapeutic responses. Notably, the integration of therapeutic modalities such as electroacupuncture (EA) with wearable monitoring technologies such as fNIRS holds strong potential for advancing individualized, feedback-informed interventions in depression care.

Building upon this premise, the present study developed a standardized electroacupuncture (EA) protocol for depression through a comprehensive review of the literature and consultation with clinical experts. A multicenter, prospective, open-label randomized controlled trial (RCT) was subsequently conducted to compare the efficacy of pharmacotherapy alone with that of pharmacotherapy combined with EA directly. Specifically, the study employed a multidimensional outcome framework encompassing traditional rating scales, behavioral cognitive tasks, and objective neurophysiological metrics derived from fNIRS. This integrative evaluation approach enables a more comprehensive understanding of treatment effects, both subjectively and biologically. By incorporating wearable neuroimaging into the clinical trial design, this study aims to generate robust evidence to optimize depression management and guide future clinical practice and policy decisions.

In summary, this study makes the following main contributions: (1) it is the first multicenter, prospective, randomized controlled trial to directly compare pharmacotherapy alone with pharmacotherapy combined with standardized EA for depression; (2) it employs a multidimensional outcome framework—including subjective rating scales, objective cognitive tasks, and fNIRS-based neurophysiological measures—to comprehensively assess treatment efficacy; (3) it demonstrates that EA combined with pharmacotherapy produces greater improvements in cognitive function and cortical activation over time, providing novel clinical and neurophysiological evidence; and (4) it highlights the feasibility of integrating wearable neuroimaging (fNIRS) into clinical trials, supporting precision-oriented and feedback-informed interventions for depression.

## 2 Materials and methods

### 2.1 Study design

**Figure 1:**
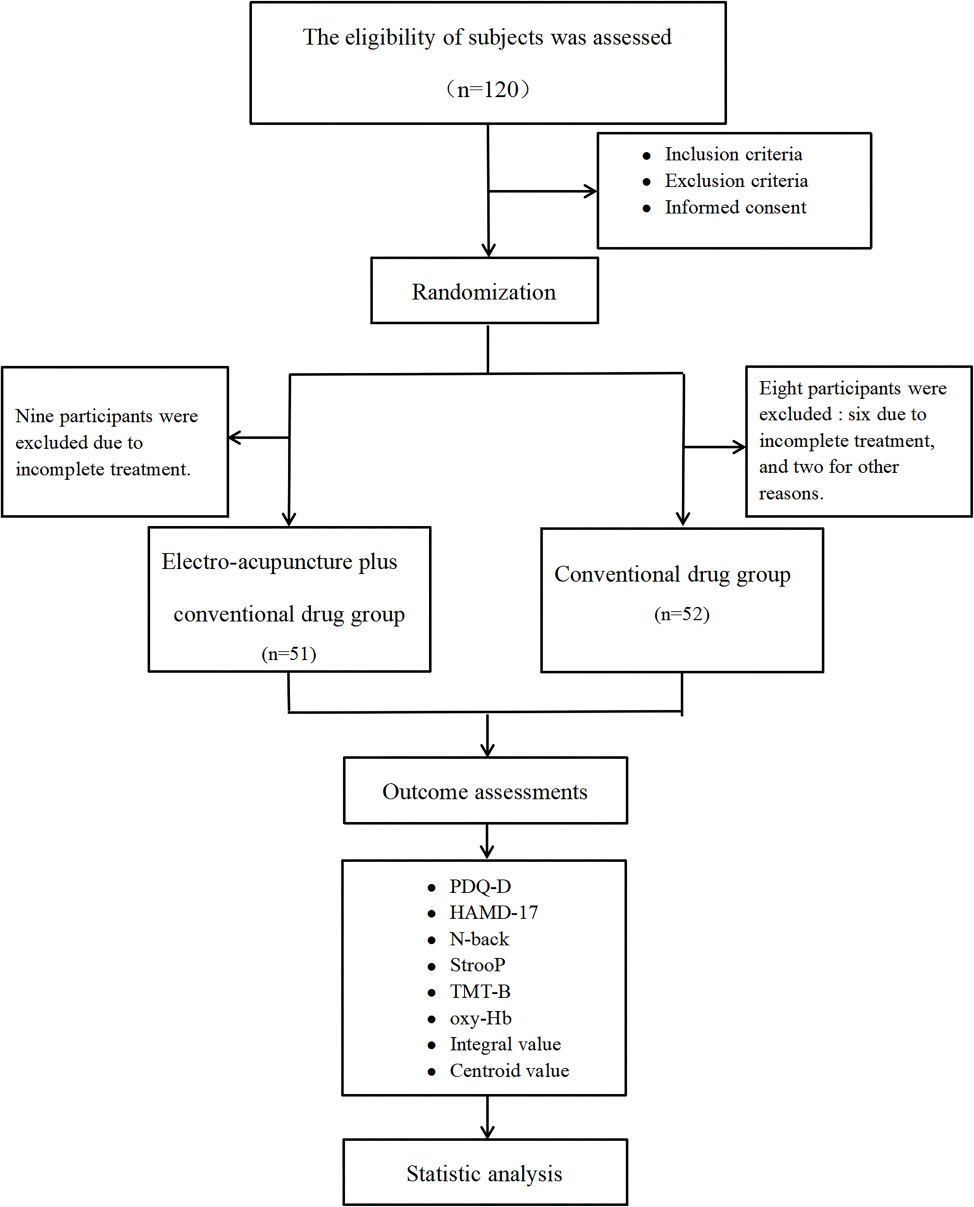
Flow chart.

Between March and December 2024, this multicenter, prospective, open-label randomized controlled trial (RCT) was conducted at two tertiary care centers: the Department of Encephalopathy II, Shaanxi Provincial Hospital of Traditional Chinese Medicine, Xi’an, China, and the Department of Psychiatry and Psychology, Yan’an University Xianyang Hospital, Xianyang, China. Participant recruitment was conducted through posters displayed at both centers. All participants provided written informed consent prior to randomization, following a standardized protocol. The study was conducted in strict compliance with the ethical principles outlined in the Declaration of Helsinki (1964) and its subsequent amendments, with oversight by the institutional review boards of both centers. Ethical approval was obtained from the Ethics Committee of Shaanxi Provincial Hospital of Traditional Chinese Medicine (Approval No. [2024] 25) and Yan’an University Xianyang Hospital (Approval No. YDXY-KY-2024-009). This trial was prospectively registered with the Chinese Clinical Trial Registry (ChiCTR2400082987). Reporting of the study adhered to the Consolidated Standards of Reporting Trials (CONSORT 2010) as well as the Standards for Reporting Interventions in Clinical Trials of Acupuncture (22, 23). **Figure 1** illustrates the trial enrollment schema.

### 2.2 Patients

The inclusion criteria were as follows: (1) Patients with either a new or previous diagnosis of major depressive disorder, based on the Diagnostic and Statistical Manual of Mental Disorders, Fifth Edition (DSM-5) criteria (24). (2) Those who scored PDQ-D ≥21, reflecting self-reported cognitive deficits. (3) No antidepressant medication use or acupuncture treatment was used within two months prior to enrollment, and no prior history of acupuncture treatment, specifically for depression, was given. (4) Those with HAMD-17 scores between 8 and 24 (25). (5) Patients who were aged between 18 and 60 years. (6) Those who provided written informed consent to participate in the study. The exclusion criteria were as follows: (1) patients with depression secondary to other severe psychiatric disorders, such as schizophrenia, bipolar disorder, and obsessive-compulsive disorder; (2) patients with a high risk for suicide or self-harm behaviors; (3) pregnant or lactating women; (4) patients who received neuromodulation treatments (such as electroconvulsive therapy, transcranial magnetic stimulation, and transcranial direct current stimulation) within 6 months before enrollment; (5) patients with a history of needle syncope; and (6) patients with skin lesions at acupuncture sites. The withdrawal and discontinuation criteria were as follows: (1) participants who failed to complete the clinical trial; (2) those who experienced adverse events preventing continuation of the study; (3) occurrence of critical safety issues during the trial; (4) identification of major protocol violations or significant deviations during the implementation of the trial; and (5) inability to ensure participant safety effectively.

### 2.3 Sample size

The calculation of sample size was based on the PDQ-D score, the primary outcome measure. The estimated means ± standard deviations (SDs) were 20.6 ± 13.5 and 15.6 ± 9.8 for the control and experimental groups, respectively. The required sample size was calculated using PASS 11.0 software with a statistical power (1-β) of 80% and a significance level (α) of 0.05. The sample size was calculated as 54 participants per group, totaling 108 participants. The final sample size was adjusted to 60 participants per group, considering an anticipated dropout rate of 10% during the trial, resulting in a total of 120 participants.

### 2.4 Randomization and blinding

The eligible patients were randomized after written informed consent was obtained. Randomization was performed using a random number table generated using R software version 4.5.0. The participants were assigned at a 1:1 ratio to either the conventional medication group or the EA combined with conventional medication group. Stratified block randomization was used, with a block size of 3. The designated research personnel managed the allocation sequence and securely stored it. After randomization of the eligible participants, researchers notified the acupuncturists of the corresponding random numbers through a short message to ensure transparency and fairness throughout the process of the study. Owing to the specific nature of acupuncture procedures, this study used an open-label design without blinding.

### 2.5 Intervention

#### 2.5.1 Control group

The participants in the control group received oral paroxetine hydrochloride tablets at an initial dose of 20 mg once daily (Manufacturer: Zhejiang Huahai Pharmaceutical Co., Ltd.; Batch No. H20031106; Specification: 20 mg × 10 tablets/strip × 2 strips/box) regularly for 4 weeks. The starting dose was 20 mg in the first week. The dose could be increased by 10 mg per week, based on the response of patients after 2 weeks, up to a maximum dose of 40 mg daily, and maintained for the complete treatment period of 4 weeks.

For patients who were intolerant to paroxetine hydrochloride, replacement with sertraline hydrochloride tablets 50 mg once every morning was permitted (Tianjin Huajin Pharmaceutical Co., Ltd.; Approval No: National Medicine Standard H20051553; Specification: 50 mg × 14 tablets/strip × 1 strip/box). The initial dose was set at 50 mg once daily during the first week, with the option to increase the dosage by 50 mg each subsequent week, not exceeding a maximum daily dose of 200 mg.

Zolpidem tartrate tablets at 3 mg per dose were allowed as temporary administration for participants experiencing intolerable insomnia during the study (manufactured by Chengdu Kanghong Pharmaceutical Group Co., Ltd.; Approval No: National Medicine Standard H20100074; Specification: 3 mg × 7 tablets/strip × 1 strip/box). If needed, a bedtime dose of 1.5 mg to 3 mg could be taken orally 2 to 5 times per week.

(2) EA plus Conventional Drug Group (Intervention Group).

**Figure 2:**
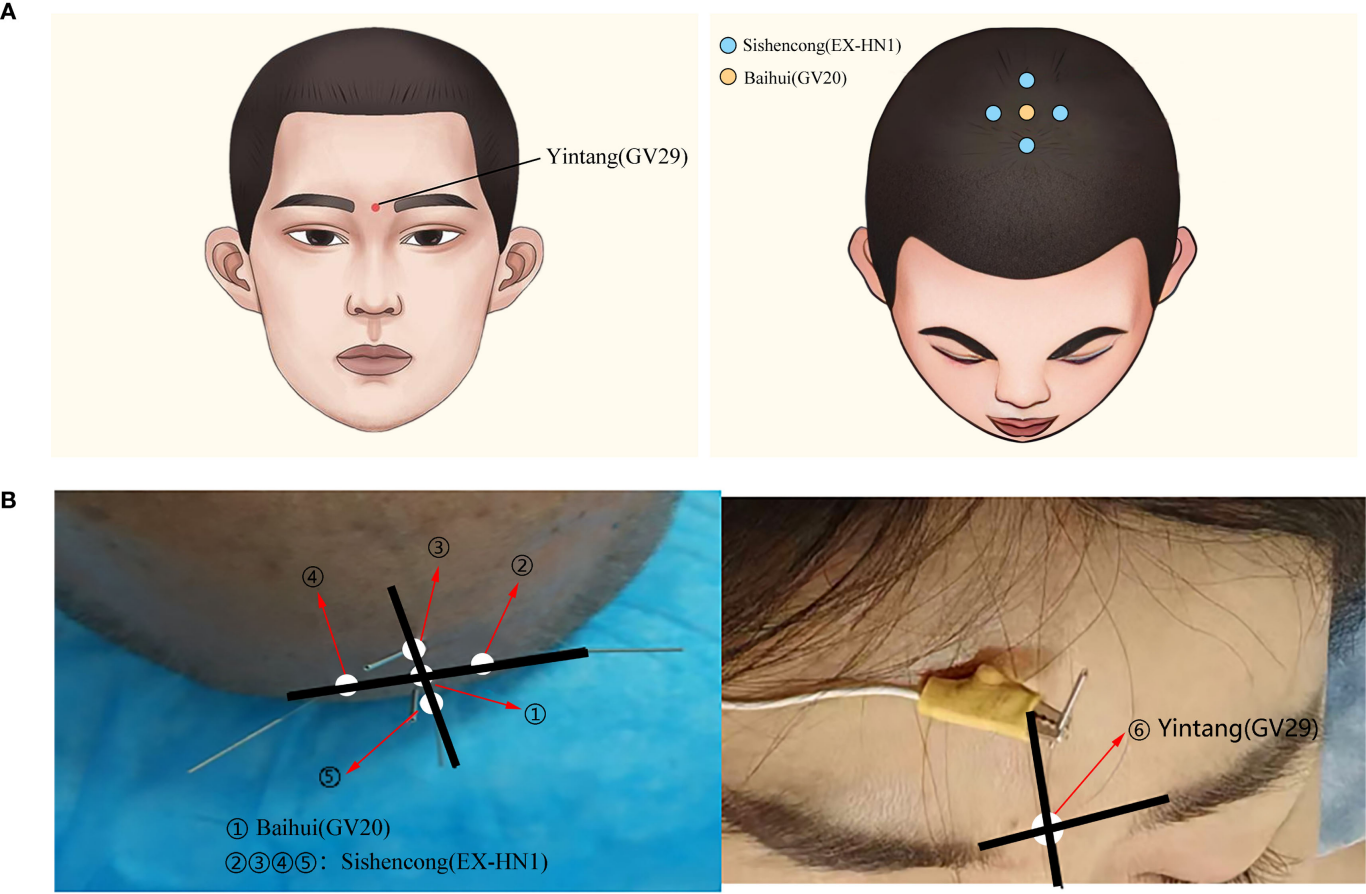
**(A)** Schematic diagram of acupoints used in EA treatment. **(B)** Clinical photographs of acupoints used in EA treatment.

The participants in the intervention group underwent EA treatment in addition to receiving the same conventional pharmacotherapy as the control group did. The selected acupoints included Baihui (GV20), Yintang (GV29), and Sishencong (EX-HN1), with detailed anatomical locations presented in **Table 1** and **Figure 2**. Sterile disposable filiform acupuncture needles (Hua Tuo brand, Suzhou Medical Supplies Factory Co., Ltd.) with 0.25 × 40 mm dimensions were used. After Deqi sensation was achieved, EA was applied via a G6802-2A EA device (Shanghai Medical Instruments Co., Ltd.), which delivered dispersed-dense wave stimulation to the Baihui and Yintang points. The stimulation pattern involved alternating dispersed and dense waves with a frequency ratio of 1:5 (1 Hz for dispersed waves and 5 Hz for dense waves). The dispersed wave operated for 5 s, followed by 9 s of dense wave operation, with a timing tolerance of ±15%. The stimulation intensity was set at level 5. The needles were retained for 30 min per session. EA was administered once daily for five consecutive days each week, followed by a two-day break, over a total period of four weeks (20 sessions in total).

**Table 1:** The locations of the acupuncture points used in the electroacupuncture group.

Acupoint	Location description
Baihui (GV20)	Located on the midline of the head, 5 cun directly above the midpoint of the anterior hairline.
Yintang (GV29)	Located at the midpoint between the medial ends of the two eyebrows.
Sishencong (EX-HN1)	Four points located 1 cun anterior, posterior, and lateral (left and right) to Baihui(GV20).

### 2.6 Outcome

**Figure 3:**
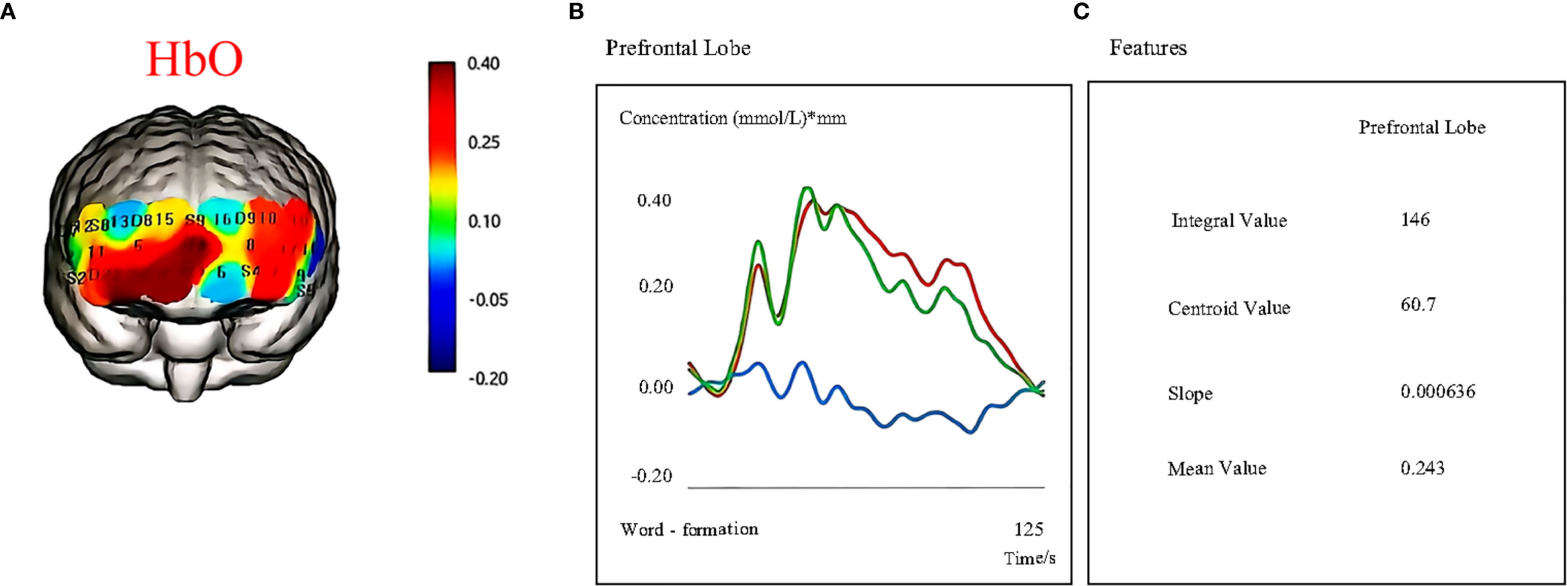
Cortical activation during cognitive task performance as measured by fNIRS. **(A)** Oxygenated hemoglobin (HbO) changes. **(B)** Hemodynamic responses (HbO, HbR, HbT) in the prefrontal cortex during task execution. **(C)** Quantitative features extracted from fNIRS signals, including the integral value, centroid value, slope, and mean value.

The primary outcome for the study was the PDQ-D (26). The secondary outcome measures included the following: HAMD-17 (27), the N-back task, the SCWT (8), and the TMT-B (28). Additionally, cerebral functional data were collected via 48-channel NirSmart-3000DS fNIRS (Huichuang Medical Technology Co., Ltd.). This system enables the extraction of indicators such as oxyhemoglobin (oxy-Hb), integral values, and centroid values. **Figure 3** shows a representative example of an fNIRS signal display during data acquisition. Safety was monitored continuously throughout the treatment and follow-up periods by recording all adverse events and other relevant clinical examination indices.

### 2.7 Quality control

#### 2.7.1 Practitioner qualifications and training

All EA treatments will be performed by licensed physicians with valid medical practitioner certificates, each with clinical acupuncture experience of more than 500 h. The practitioners will undergo standardized training before the initiation of the trial and must pass a competency assessment tailored to this protocol. Strict adherence to the study manual procedures is mandatory.

#### 2.7.2 Protocol adherence and patient eligibility

Strict enforcement of eligibility criteria is required to minimize risks related to patient noncompliance and practitioner variability. Only patients who fulfill the indications of approved medications or established clinical guidelines will be enrolled; off-label or beyond-indication medication use is generally discouraged within this protocol.

#### 2.7.3 Informed decision-making

All participants will be completely informed regarding potential adverse effects, including drug allergies, side effects, and reactions related to needles, such as syncope, and must voluntarily consent to participate in this study.

#### 2.7.4 Participant education and family support

At the time of enrollment, participants will receive a comprehensive patient handbook that outlines the benefits, responsibilities, and procedures of the study. Family members also receive health education to increase support at home, promote adherence, and facilitate smooth trial implementation.

### 2.8 Statistical analysis

R version 4.5.0 was used for the statistical analyses. Categorical variables are represented as frequencies and percentages and were compared using chi-square tests. Continuous variables were assessed for normality; normally distributed data are presented as the means ± standard deviations and were assessed using independent-samples t tests. In contrast, non-normally distributed data are reported as medians (interquartile ranges) and were compared sing the Mann-Whitney U test. The linear mixed-effects models were used with “time” and “group” as fixed effects and participant ID as a random effect to assess intervention effects over time. Covariates such as age and sex were included to control for confounding factors. All tests were two-tailed, and P < 0.05 was considered statistically significant.

## 3 Results

### 3.1 Participant recruitment and disposition

A total of 120 eligible participants were screened between March and December 2024 and randomized equally into the control and the EA plus conventional pharmacotherapy groups. In the intervention arm, because of the inability to complete treatment or assessments, nine participants withdrew; in the control arm, six withdrew owing to poor compliance, and two self-administered additional medications were withdrawn because of clinical instability. A total of 103 participants completed the trial, with 51 in the EA plus pharmacotherapy group and 52 in the control group.

### 3.2 Baseline characteristics

No statistically significant differences in sex, age, duration of illness, or other baseline characteristics were detected between the two groups (P > 0.05) (**Table 2**).

**Table 2:** Patient characteristics at baseline.

Variable	Control group (n = 52)	Intervention group (n = 51)	P value
Age	42.50 (34.50,50.50)	41.00 (30.00,50.00)	0.62
Duration(month)	7.00 (6.00,8.00)	7.00 (6.00,8.00)	0.23
Sex			0.35
Male	12 (23.08)	17 (33.33)	
Female	40 (76.92)	34 (66.67)	

*Intervention Group = Electro-acupuncture plus Conventional Drug Group.

### 3.3 Primary outcomes

#### 3.3.1 Effects of the two treatment modalities on PDQ-D scores

**Figure 4:**
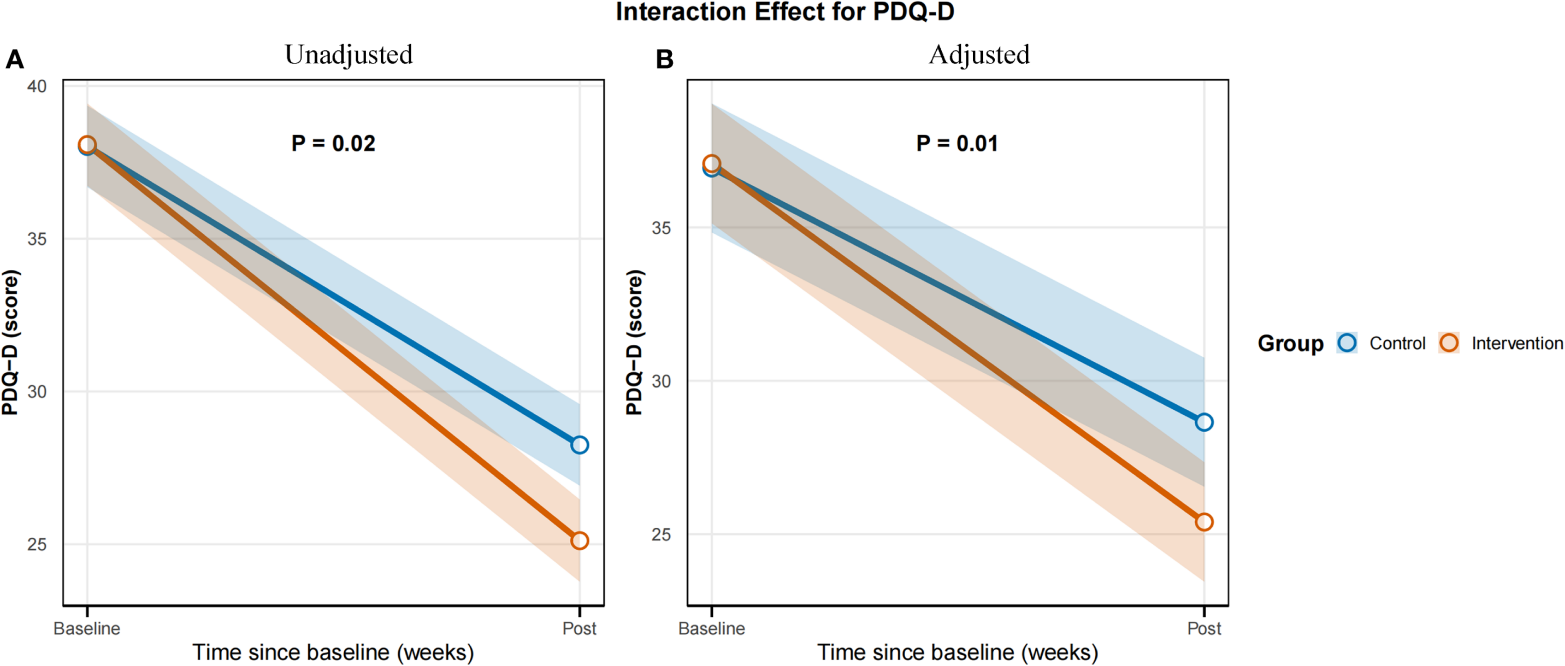
Time × group interaction effect for the primary outcome PDQ-D Scores. **(A)** Unadjusted model. **(B)** Adjusted model.

Baseline PDQ-D scores did not differ significantly between the control (38.02 ± 5.70) and EA plus pharmacotherapy groups (38.08 ± 5.51; P = 0.96), indicating comparable baseline cognitive function. Post-intervention, the EA plus pharmacotherapy group demonstrated significantly lower PDQ-D scores (25.00 [22.00, 27.50]) than the control group (29.00 [24.75, 31.25]; mean difference -3.40, 95% CI-6.10 to -0.74, P < 0.01). Linear mixed-effects model analysis revealed a significant main effect of time (P < 0.01), while the main effect of group was non-significant (P = 0.95), indicating that overall scores decreased over time but were comparable between groups if time was not considered. Importantly, the time × group interaction was significant (P = 0.02; adjusted P = 0.01), suggesting that although baseline scores were similar, the trajectory of improvement over time differed between groups, with the EA combination group showing greater improvement (**Table 3**). The interaction effect plot for the primary outcome, PDQ-D Scores, is presented in **Figure 4**.

**Table 3:** Primary outcomes.

Variables	Time point	Control group vs intervention group			Mean difference (95%CI)	Time effect (P value)	Group effect (P value)	Interaction effects (P value)	Adjust interaction effects (P value)
		Control group	Intervention group	*P* value					
PDQ-D	Baseline	38.02 ± 5.702	38.08 ± 5.51	0.96	–	<0.01	0.95	0.02	0.01
	Post-test	29.00 (24.75,31.25)	25.00 (22.00,27.50)	<0.01	-3.40(–6.10, –0.74)				

*Intervention Group = Electro-acupuncture plus Conventional Drug Group.

### 3.4 Secondary outcomes

#### 3.4.1 Effects of the two treatment modalities on HAMD-17 scores

Baseline HAMD-17 scores were comparable between the control and EA plus pharmacotherapy groups (P = 0.31) Post-intervention, the EA plus pharmacotherapy group had lower scores than the control group (mean difference -2.73, 95% CI-3.83 to -1.63, P < 0.01), showing greater symptom improvement. Linear mixed-effects analysis revealed a significant time effect (P < 0.01) and a significant time × group interaction (P < 0.01), while the main group effect was not significant (P = 0.23). The interaction remained significant after adjusting for age and sex (adjusted P < 0.01), confirming that although baseline scores were similar, the EA combination group improved more rapidly over time (**Table 4**).

**Table 4:** Secondary outcomes.

Variables	Time point	Control group vs intervention group			Mean difference (95%CI)	Time effect (P value)	Group effect (P value)	Interaction effects (P value)	Adjust interaction effects (P value)
		Control group	Intervention group	*P* value					
HAMD-17	Baseline	17.00 (15.00,19.00)	17.00 (16.00,19.50)	0.31	–	<0.01	0.23	<0.01	<0.01
	Post-test	10.00 (10.00,12.00)	8.00 (8.00,9.00)	<0.01	-2.73 (–3.83, –1.63)				
N-back accuracy	Baseline	64.06 ± 3.37	63.10 ± 3.38	0.15	–	<0.01	0.13	<0.01	<0.01
	Post-test	80.00 (78.00,82.00)	90.00 (88.00,91.50)	<0.01	10.01 (8.34,11.68)				
N-back Reaction Time	Baseline	887.00 (785.75,924.25)	879.00 (837.50,921.50)	0.84	–	<0.01	0.97	<0.01	<0.01
	Post-test	648.13 ± 75.09	526.12 ± 30.45	<0.01	-118.23 (–166.51, –69.96)				
SCWT accuracy	Baseline	74.00 (72.00,76.25)	75.00 (72.50,78.00)	0.19	–	<0.01	0.27	<0.01	<0.01
	Post-test	84.00 (82.00,87.00)	95.00 (94.00,97.00)	<0.01	9.38 (7.02,11.73)				
SCWT Reaction Time	Baseline	929.00 (847.50,1016.00)	912.00 (834.50,1017.00)	0.78	–	<0.01	0.91	0.01	<0.01
	Post-test	679.00 (607.50,779.50)	574.00 (515.00,699.00)	<0.01	-84.35 (–143.99, –24.69)				
TMT-B accuracy	Baseline	71.65 ± 3.52	71.55 ± 3.40	0.88	–	<0.01	0.88	<0.01	<0.01
	Post-test	80.79 ± 3.49	89.10 ± 3.27	<0.01	8.31 (6.42,10.20)				
TMT-B Reaction Time	Baseline	128.79 ± 11.52	128.24 ± 11.97	0.81	–	<0.01	0.80	<0.01	<0.01
	Post-test	97.50 (91.00,106.25)	87.00 (81.00,92.50)	<0.01	-10.50 (–16.39, –4.60)				
fNIRS									
oxy-Hb	Baseline	0.03 ± 0.01	0.03 ± 0.01	0.25	–	<0.01	0.54	<0.01	<0.01
	Post-test	0.11 (0.10,0.12)	0.19 (0.17,0.21)	<0.01	0.08 (0.07,0.09)				
Integral value	Baseline	28.88 ± 2.89	29.10 ± 2.61	0.69	–	<0.01	0.13	<0.01	<0.01
	Post-test	42.50 (39.50,45.50)	57.50 (56.35,59.60)	<0.01	15.07 (13.54,16.61)				
Centroid value	Baseline	68.00 (66.00,69.50)	68.50 (67.25,70.75)	0.06	–	<0.01	0.01	<0.01	<0.01
	Post-test	46.12 ± 2.79	32.43 ± 2.98	<0.01	-16.87 (–20.56, –13.18)				

* Intervention Group = Electro-acupuncture plus Conventional Drug Group

#### 3.4.2 Effects of the two treatment modalities on N-back performance

Baseline N-back accuracy (P = 0.15) and reaction time (P = 0.84) were comparable between groups, indicating similar baseline working memory. Post-intervention, the EA plus pharmacotherapy group achieved higher accuracy (mean difference 10.01, 95% CI 8.34 – 11.68, P < 0.01) and shorter reaction times (mean difference -118.23, 95% CI-166.51 to -69.96, P < 0.01) compared with controls. Mixed-effects analysis revealed significant time effects and time × group interactions for both measures (all P < 0.01), but no main group effects (accuracy: P = 0.13; reaction time: P = 0.97). These interactions remained significant after adjusting for age and sex (all adjusted P < 0.01), confirming that although baseline performance was similar, the EA group showed greater improvements in working memory and processing speed over time (**Table 4**).

#### 3.4.3 Effects of the two treatment modalities on SCWT

SCWT accuracy and reaction time were comparable between groups at baseline (P = 0.19 and 0.78, respectively). Post-intervention, the EA plus pharmacotherapy group achieved significantly higher accuracy (mean difference = 9.38, 95% CI 7.02 – 11.73, P < 0.01) and faster reaction times (mean difference = –84.35, 95% CI – 143.99 to –24.69, P < 0.01). Mixed-effects analysis revealed significant time effects and time ×group interactions for both measures (all P < 0.01), without significant main group effects (accuracy: P = 0.27; reaction time: P = 0.91). These interactions remained significant after adjusting for age and sex (all adjusted P < 0.01), confirming that although baseline performance was comparable, the EA group showed greater improvements in selective attention and executive control over time (**Table 4**).

#### 3.4.4 Effects of the two treatment modalities on TMT-B performance

At baseline, no significant differences were detected between groups in TMT-B accuracy (P = 0.88) or completion time (P = 0.81), indicating comparable cognitive flexibility and processing speed. Post-intervention, the EA plus pharmacotherapy group was found to have significantly higher accuracy (mean difference = 8.31, 95% CI 6.42 – 10.20, P < 0.01) and shorter completion times (mean difference = –10.50, 95% CI – 16.39 to –4.60, P < 0.01) than the control group. Significant main effects of time and time ×group interactions were revealed using mixed-effects modeling for both outcomes (all P < 0.01), with non-significant group effects (accuracy: P = 0.88; time: P = 0.80). After adjusting for age and sex, the interaction effects remained significant (all adjusted P ≤ 0.01), confirming that although baseline performance was comparable, greater improvements in executive function were achieved in the EA plus pharmacotherapy group over time (**Table 4**).

#### 3.4.5 Effects of the two treatment modalities on fNIRS parameters

At baseline, no significant differences were detected between groups in fNIRS-derived measures, including oxy-Hb concentration, integral value, and centroid value (all P > 0.05), confirming comparability. Post-intervention, significantly greater improvements across all the parameters were observed in the EA plus pharmacotherapy group (all P < 0.01). Mixed-effects model analysis revealed significant time effects and time × group interactions for oxy-Hb concentration and integral values (both P < 0.01), with non-significant group main effects (P = 0.54 and P = 0.13, respectively). These results indicated that oxy-Hb concentration and integral values increased more markedly over time in the EA group, reflecting enhanced cortical activation and sustained hemodynamic responses. For the centroid value, significant effects of group, time, and their interaction were detected (all P < 0.01), and these differences remained statistically significant after adjusting for age and sex (P < 0.01), suggesting that centroid values declined more rapidly over time in the EA group, reflecting more efficient neural processing (**Table 4**).

#### 3.4.6 Adverse events

Throughout the study period, no adverse events, such as needle retention, needle breakage, or bleeding, occurred in the intervention group. Additionally, all clinical laboratory indices remained within normal limits in both the acupuncture and control groups.

## 4 Discussion

Depression, a chronic psychiatric disorder that includes affective, somatic, and cognitive disturbances, significantly decreases quality of life and poses a major global health burden (29, 30). Although pharmacotherapy is the primary treatment for depression, it has limited effects on cognitive impairment. Consequently, interest in adjunctive therapies has increased. This study assessed the efficacy of combining EA with standard pharmacotherapy, with a particular focus on improving cognitive function in patients with depression.

Our findings demonstrate that both standard pharmacotherapy alone and its combination with EA provide therapeutic benefits. Crucially, compared with pharmacotherapy alone, adjunctive EA demonstrated superior efficacy across multiple domains. These benefits included significant improvements in cognitive deficits, a reduction in depressive symptoms, increased memory capacity, and gains in cognitive flexibility, selective attention, and task execution. In support of these neuropsychological outcomes, fNIRS assessments revealed that the combined treatment group presented superior cortical activation patterns, characterized by significantly greater increases in oxyhemoglobin concentration, integral values, and centroid values.

These results align with prior clinical evidence suggesting that EA in combination with pharmacotherapy results in faster onset and safer antidepressant effects than does medication alone. For example, Zhao, B. al (31) demonstrated that adjunctive EA targeting Baihui (GV20), Yintang (GV29), Fengfu (GV16), bilateral Fengchi (GB20), Dazhui (GV14), bilateral Neiguan (PC6), and bilateral Sanyinjiao (SP6) significantly improved cognitive symptoms in patients with depression compared with medication alone. Although the acupoints used in that study differed from those used in the present study, both sets of acupoints share similar therapeutic properties, such as calming the mind, restoring consciousness, regulating spirit, and unblocking meridians to relieve pain. Similarly, Zhang et al., through a more comprehensive meta-analysis, further confirmed that EA combined with selective serotonin reuptake inhibitors (SSRIs) provided significantly greater symptom relief and better safety profiles than SSRIs alone during the early phase of treatment (weeks 1 – 2). The analysis also indicated that, whether used as monotherapy or in combination with SSRIs, EA was not inferior to SSRIs alone and often demonstrated superior efficacy, with faster onset and fewer adverse effects (32). However, research specifically targeting cognitive symptoms in depression remains limited, and findings are sometimes inconsistent. A recent pilot RCT [29] revealed that compared with sham EA plus standard care, EA improved subjective cognitive complaints in major depressive disorder (MDD) patients but did not yield significant effects on objective cognitive domains, quality of life, or depressive severity. This discrepancy may reflect methodological variations in assessment, intervention protocols, or sample characteristics (33).

EA has also demonstrated significant potential in improving cognitive impairment associated with other neurological disorders, such as Alzheimer’s disease (34), Parkinson’s disease (35), and mild cognitive impairment (MCI) (36). A systematic review and meta-analysis encompassing five randomized controlled trials revealed that compared with conventional pharmacological treatments, EA moderately enhanced patients’ performance on the Mini-Mental State Examination (MMSE) and the Montreal Cognitive Assessment (MoCA) (37). Underlying neurobiological mechanisms are supported by animal studies, which have demonstrated that EA can alleviate depression-like behaviors and cognitive deficits in rodents, potentially through suppressing oxidative stress-induced neuroinflammation and promoting neuronal repair (38).

A key strength of our study lies in providing neuroimaging-level evidence via fNIRS. While previous acupuncture neuroimaging studies in individuals with depression have reported activation changes in regions such as the frontopolar cortex or dorsolateral prefrontal cortex (DLPFC), few studies have quantitatively analyzed detailed hemodynamic metrics such as integral or centroid values (39). Our work uniquely demonstrated that adjunctive EA significantly modulates cortical oxygenation and hemodynamic dynamics in target brain regions and that these changes correlate with the observed cognitive improvements.

Methodologically, we employed a multimodal framework integrating neuropsychological assessments (HAMD-17, N-back, SCWT, TMT-B) with functional near-infrared spectroscopy (fNIRS) imaging, complementing outcomes from the Perceived Deficits Questionnaire for Depression (PDQ-D). The adjunctive electroacupuncture (EA) group demonstrated consistent superiority over the control group across all secondary endpoints, aligning with self-reported cognitive improvements. These findings suggest a synergistic “symptom-cognition” recovery mechanism. We hypothesize that EA enhances top-down regulation within fronto-limbic circuits while simultaneously promoting hippocampal neuroplasticity via bottom-up processes, thereby facilitating the concurrent restoration of emotional and cognitive function (40). Collectively, the convergence of subjective reports, objective cognitive performance, and neuroimaging evidence supports the clinical utility of EA as a viable adjunctive intervention for depression and provides mechanistic insights into its underlying neurobiology.

Despite these promising findings, this study has several limitations that warrant consideration. First, the relatively small sample size (N = 103) may have reduced the statistical power to detect smaller effect sizes, thereby limiting the generalizability of the results. Second, the study lacked a sham EA control group, which restricts the ability to distinguish the specific effects of EA from potential placebo responses or expectancy biases. Third, although all participants received standard pharmacotherapy, changes in medication dosage or adherence were not quantitatively monitored throughout the study period. This omission may confound the interpretation of EA’ s independent contribution to the observed outcomes. Future research should implement dynamic tracking of medication use to more accurately isolate the intervention’ s effects. Finally, the intervention duration was relatively short (four weeks), which may not have been sufficient to capture the full scope of the therapeutic potential of EA, particularly with respect to long-term cognitive and emotional recovery. Longer treatment durations and extended follow-up assessments are recommended. Future studies should also explore the neurobiological mechanisms underlying EA’ s effects using multimodal neuroimaging and molecular techniques, optimize stimulation parameters for individualized therapy, and integrate wearable monitoring technologies to enable real-time, feedback-informed interventions. Such approaches could help translate EA into more precise and personalized treatment strategies for cognitive impairment in depression.

Importantly, these limitations notwithstanding, the current findings demonstrate that EA combined with standard pharmacotherapy holds promise as a safe, well-tolerated, and clinically applicable adjunctive therapy for cognitive impairment in depression, providing a foundation for further in-depth trials and the development of personalized, feedback-informed interventions.

## 5 Conclusion

In this study, subjective rating scales, objective behavioral tasks, and neuroimaging indices were integrated to comprehensively assess the effects of EA combined with standard pharmacotherapy on cognitive impairment in patients with depression. The results of the present study show that the combined intervention yields superior improvements in cognitive function than pharmacotherapy alone. EA plus medication shows promising clinical applicability as a safe and well-accepted adjunctive therapy. These findings support the potential of this multimodal strategy and establish a robust foundation for further in-depth trials.

## Data Availability

The raw data supporting the conclusions of this article will be made available by the authors, without undue reservation.

## References

[1] Diseases GBD Injuries C. Global incidence, prevalence, years lived with disability (YLDs), disability-adjusted life-years (DALYs), and healthy life expectancy (HALE) for 371 diseases and injuries in 204 countries and territories and 811 subnational locations, 1990-2021: a systematic analysis for the Global Burden of Disease Study 2021. Lancet. (2024) 403:2133–61. doi: 10.1016/S0140-6736(24)00757-8, PMID: 38642570 PMC11122111

[2] Collaborators GBDMD. Global, regional, and national burden of 12 mental disorders in 204 countries and territories, 1990-2019: a systematic analysis for the Global Burden of Disease Study 2019. Lancet Psychiatry. (2022) 9:137–50. doi: 10.1016/S2215-0366(21)00395-3, PMID: 35026139 PMC8776563

[3] Barnett R. Depression. Lancet. (2019) 393:2113. doi: 10.1016/S0140-6736(19)31151-1, PMID: 31226037

[4] Herrman H Patel V Kieling C Berk M Buchweitz C Cuijpers P. Time for united action on depression: a Lancet-World Psychiatric Association Commission. Lancet. (2022) 399:957–1022. doi: 10.1016/S0140-6736(21)02141-3, PMID: 35180424

[5] Bayes AJ Parker GB. Comparison of guidelines for the treatment of unipolar depression: a focus on pharmacotherapy and neurostimulation. Acta Psychiatr Scand. (2018) 137:459–71. doi: 10.1111/acps.12878, PMID: 29577229

[6] Ren X Zhao X Li J Liu Y Ren Y Pruessner JC. The hippocampal-ventral medial prefrontal cortex neurocircuitry involvement in the association of daily life stress with acute perceived stress and cortisol responses. Psychosom Med. (2022) 84:276–87. doi: 10.1097/PSY.0000000000001058, PMID: 35149637

[7] Lv Q Li X Zhang Y Lu D Lu J Xie Q. Sex differences in subjective cognitive impairment and clinical correlates in Chinese patients with subthreshold depression. Biol Sex Differ. (2023) 14:6. doi: 10.1186/s13293-023-00488-w, PMID: 36782299 PMC9926784

[8] Alcon C Krieger C Neal K. The relationship between pain catastrophizing, kinesiophobia, central sensitization and cognitive function in patients with chronic low back pain. Clin J Pain. (2025) 41(7). doi: 10.1097/AJP.0000000000001293, PMID: 40261050

[9] Ou A Wu GWY Kassel MT Mackin RS Rampersaud R Reus VI. Cognitive function in physically healthy, unmedicated individuals with major depression: Relationship with depressive symptoms and antidepressant response. J Affect Disord. (2025) 378:191–200. doi: 10.1016/j.jad.2025.02.115, PMID: 40032138

[10] Khan A Mar KF Brown WA. The conundrum of depression clinical trials: one size does not fit all. Int Clin Psychopharmacol. (2018) 33:239–48. doi: 10.1097/YIC.0000000000000229, PMID: 29939890 PMC6078483

[11] Wang X Zhou J Zhu K Wang Y Ma X Ren L. Efficacy and safety of Neurocognitive Adaptive Training for Depression combined with SSRIs for treating cognitive impairment among patients with late-life depression: a 12-week, randomized controlled study. BMC Psychiatry. (2024) 24:848. doi: 10.1186/s12888-024-06276-z, PMID: 39587504 PMC11590405

[12] Chan YY Lo WY Yang SN Chen YH Lin JG. The benefit of combined acupuncture and antidepressant medication for depression: A systematic review and meta-analysis. J Affect Disord. (2015) 176:106–17. doi: 10.1016/j.jad.2015.01.048, PMID: 25704563

[13] Wang H Qi H Wang BS Cui YY Zhu L Rong ZX. Is acupuncture beneficial in depression: a meta-analysis of 8 randomized controlled trials? J Affect Disord. (2008) 111:125–34., PMID: 18550177 10.1016/j.jad.2008.04.020

[14] Xia J Jiang M Yin X Wang Z Li F Wei H. Efficacy and safety of electroacupuncture on treating mild to moderate first-episode depression: a study protocol for a randomized controlled trial. Front Psychiatry. (2025) 16:1521859. doi: 10.3389/fpsyt.2025.1521859, PMID: 40078528 PMC11897229

[15] Boyang Z Yang Z Liyuan F Dan S Lei HE Dan T. A neural regulation mechanism of head electroacupuncture on brain network of patients with stroke related sleep disorders. J Tradit Chin Med. (2024) 44:1268–76., PMID: 39617712 10.19852/j.cnki.jtcm.2024.06.011 PMC11589561

[16] Li W Sun M Yin X Lao L Kuang Z Xu S. The effect of acupuncture on depression and its correlation with metabolic alterations: A randomized controlled trial. Med (Baltimore). (2020) 99:e22752. doi: 10.1097/MD.0000000000022752, PMID: 33120777 PMC7581113

[17] Bin Heyat MB Adhikari D Akhtar F Parveen S Zeeshan HM Ullah H. Intelligent internet of medical things for depression: current advancements, challenges, and trends. Int J Intelligent Syst. (2025) 2025.

[18] Bin Heyat MB Akhtar F Abbas SJ Al-Sarem M Alqarafi A Stalin A. Wearable flexible electronics based cardiac electrode for researcher mental stress detection system using machine learning models on single lead electrocardiogram signal. Biosensors (Basel). (2022) 12(6):427. doi: 10.3390/bios12060427, PMID: 35735574 PMC9221208

[19] Pal R Adhikari D Heyat MBB Guragai B Lipari V Brito Ballester J. A novel smart belt for anxiety detection, classification, and reduction using IIoMT on students’ Cardiac signal and MSY. Bioengineering (Basel). (2022) 9(12):793. doi: 10.3390/bioengineering9120793, PMID: 36550999 PMC9774730

[20] Buele J Palacios-Navarro G. Cognitive-motor interventions based on virtual reality and instrumental activities of daily living (iADL): an overview. Front Aging Neurosci. (2023) 15:1191729. doi: 10.3389/fnagi.2023.1191729, PMID: 37396651 PMC10311491

[21] Yang H Teng J Qian Y Huang T Dong M Wang H. Cortical hemodynamic abnormalities associated with fine motor deficits in mild cognitive impairment. CNS Neurosci Ther. (2025) 31:e70547. doi: 10.1111/cns.70547, PMID: 40726141 PMC12304437

[22] Schulz KF Altman DG Moher D Group C. CONSORT 2010 statement: updated guidelines for reporting parallel group randomised trials. PloS Med. (2010) 7:e1000251. doi: 10.1371/journal.pmed.1000251, PMID: 20352064 PMC2844794

[23] MacPherson H Altman DG Hammerschlag R Youping L Taixiang W White A. Revised STandards for reporting interventions in clinical trials of acupuncture (STRICTA): extending the CONSORT statement. J Evid Based Med. (2010) 3:140–55. doi: 10.1111/j.1756-5391.2010.01086.x, PMID: 21349059

[24] Park SC Kim YK. Challenges and strategies for current classifications of depressive disorders: proposal for future diagnostic standards. Adv Exp Med Biol. (2021) 1305:103–16. doi: 10.1007/978-981-33-6044-0_7, PMID: 33834397

[25] Hamilton M. A rating scale for depression. J Neurol Neurosurg Psychiatry. (1960) 23:56–62. doi: 10.1136/jnnp.23.1.56, PMID: 14399272 PMC495331

[26] Rosso G Porceddu G Portaluppi C Garrone C Di Salvo G Maina G. Exploring cognitive symptoms in patients with unipolar and bipolar major depression: A comparative evaluation of subjective and objective performance. Psychiatry Res. (2025) 347:116422. doi: 10.1016/j.psychres.2025.116422, PMID: 40023095

[27] Zhou J Xu J Feng Z Liu R Xiao L Li R. A longitudinal analysis of the relationship between emotional symptoms and cognitive function in patients with major depressive disorder. Psychol Med. (2025) 55:e131. doi: 10.1017/S0033291725001011, PMID: 40314169 PMC12094654

[28] De Anda-Duran I Hwang PH Drabick DA Andersen SL Au R Libon DJ. Neuropsychological phenotypic characteristics in a cohort of community-based older adults: Data from the Framingham Heart Study. J Alzheimers Dis. (2025) 2025:13872877251334608. doi: 10.1177/13872877251334608, PMID: 40267270

[29] Diseases GBD Injuries C. Global burden of 369 diseases and injuries in 204 countries and territories, 1990-2019: a systematic analysis for the Global Burden of Disease Study 2019. Lancet. (2020) 396:1204–22. doi: 10.1016/S0140-6736(20)30925-9, PMID: 33069326 PMC7567026

[30] Zhou J Xu J Liu R Qi H Yang J Guo T. A prospective cohort study of depression (PROUD) in China: rationale and design. Curr Med (Cham). (2023) 2:1. doi: 10.1007/s44194-022-00018-7, PMID: 36643216 PMC9826756

[31] Zhao B Li Z Wang Y Ma X Wang X Wang X. Manual or electroacupuncture as an add-on therapy to SSRIs for depression: A randomized controlled trial. J Psychiatr Res. (2019) 114:24–33. doi: 10.1016/j.jpsychires.2019.04.005, PMID: 31015098

[32] Zhang Z Cai X Liang Y Zhang R Liu X Lu L. Electroacupuncture as a rapid-onset and safer complementary therapy for depression: A systematic review and meta-analysis. Front Psychiatry. (2022) 13:1012606. doi: 10.3389/fpsyt.2022.1012606, PMID: 36684018 PMC9853905

[33] Boontra Y Thanetnit C Phanasathit M. Effects of electroacupuncture on cognitive symptoms in major depressive disorder: a pilot study and randomized controlled trial. F1000Res. (2024) 13:479. doi: 10.12688/f1000research, PMID: 39620153 PMC11605169

[34] Choi Y Kim PW Jung IC Kim AR Park HJ Kwon O. Acupuncture for patients with mild cognitive impairment: a randomized, patient-assessor-blinded, sham-controlled pilot study. BMC Complement Med Ther. (2025) 25:277. doi: 10.1186/s12906-025-05023-5, PMID: 40684196 PMC12275449

[35] Hong Z Zhang S Zhang S Zhao Y Ye X Shu X. Effect of acupuncture on the gait disturbance and hemodynamic changes in the prefrontal cortex: a study protocol for a randomized controlled trial. Front Neurol. (2024) 15:1444873. doi: 10.3389/fneur.2024.1444873, PMID: 39882359 PMC11777019

[36] Choi Y Jung IC Kim AR Park HJ Kwon O Lee JH. Feasibility and effect of electroacupuncture on cognitive function domains in patients with mild cognitive impairment: A pilot exploratory randomized controlled trial. Brain Sci. (2021) 11(6):756. doi: 10.3390/brainsci11060756, PMID: 34200354 PMC8228462

[37] Kim H Kim HK Kim SY Kim YI Yoo HR Jung IC. Cognitive improvement effects of electro-acupuncture for the treatment of MCI compared with Western medications: a systematic review and Meta-analysis. BMC Complement Altern Med. (2019) 19:13. doi: 10.1186/s12906-018-2407-2, PMID: 30621676 PMC6325879

[38] Tong T Hao C Shen J Liu S Yan S Aslam MS. Electroacupuncture ameliorates chronic unpredictable mild stress-induced depression-like behavior and cognitive impairment through suppressing oxidative stress and neuroinflammation in rats. Brain Res Bull. (2024) 206:110838. doi: 10.1016/j.brainresbull.2023.110838, PMID: 38123022

[39] Wu H Lu B Xiang N Qiu M Da H Xiao Q. Different activation in dorsolateral prefrontal cortex between anxious depression and non-anxious depression during an autobiographical memory task: A fNIRS study. J Affect Disord. (2024) 362:585–94. doi: 10.1016/j.jad.2024.07.031, PMID: 39019227

[40] Wang Y Du X Duan C Wang M Zhu Y Wang L. Regulating the plasticity of hippocampal neurons via electroacupuncture in depression model mice. Cell Prolif. (2025) 2025:e70057. doi: 10.1111/cpr.70057, PMID: 40374592 PMC12508685

