# Supplementary material for: Exploring the neural mechanisms of electroacupuncture for cognitive impairment in depression using functional near-infrared spectroscopy: a randomized controlled trial
Source: Front Psychiatry. 2025 Sep 24;16:1650695. doi: 10.3389/fpsyt.2025.1650695 (PMC12504231; doi:10.3389/fpsyt.2025.1650695)
Supplement: Supplementary file 1 [file DataSheet1.pdf]

1、 Figure 1. Mixed-effects model analysis of the intervention effect on N-back accuracy. Comparison of the time × group interaction before and after covariate adjustment. The x-axis represents follow-up time (baseline to 4 weeks post-intervention, labeled "Post-intervention"), and the y-axis indicates N-back accuracy. In the unadjusted model (Fig. 1A), The intervention significantly enhanced N-back accuracy regardless of covariate adjustment (P < 0.01), The effect was more robust after controlling for covariates, The intervention exerts independent therapeutic effects on symptom improvement. These results indicate the intervention significantly improved N-back accuracy, and this effect was stronger after adjusting for covariates, confirming its independent therapeutic benefit.

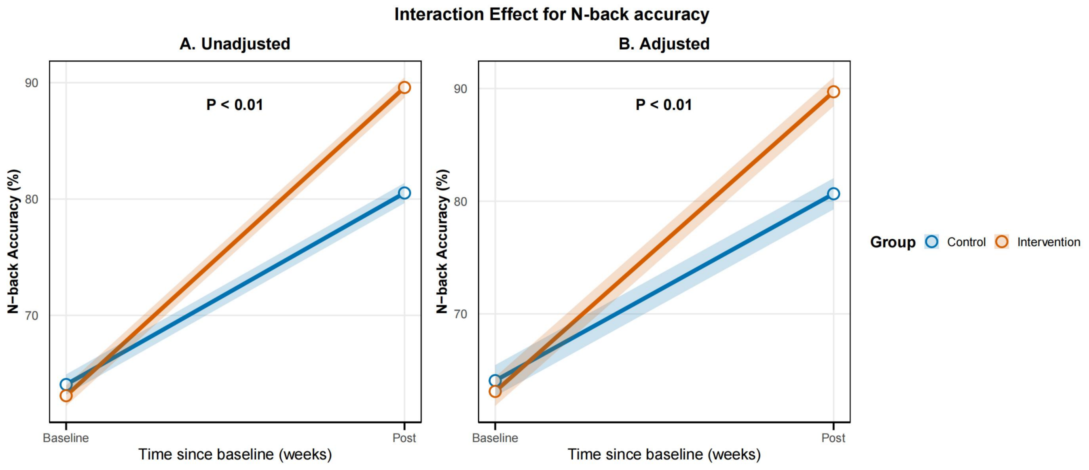


Figure 1 Mixed-effects model analysis of N-back accuracy. The plots compare the time × group interaction effects before (A) and after (B) adjustment for covariates.

2、 Figure 2 shows mixed-effects model analysis of the intervention’s effect on N-back reaction time. The x-axis is follow-up time (baseline to 4 weeks post-intervention). In the unadjusted model (Fig. 2A), a significant time × group interaction was found (P < 0.01), with the intervention group showing greater reduction in reaction time compared to controls. After adjusting for covariates (Fig. 2B), this effect was even stronger (P < 0.01), indicating faster improvement in the intervention group. These results confirm the intervention significantly enhances cognitive processing speed, with effects remaining robust after controlling for confounders, demonstrating independent therapeutic benefits.

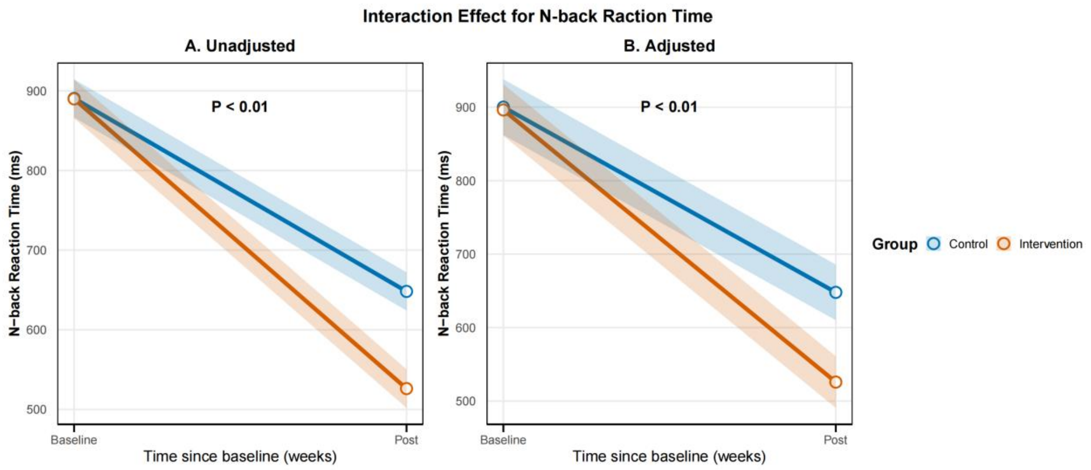


Figure 2 Mixed-effects model analysis of N-back reaction time. The plots compare the time × group interaction effects before (A) and after (B) adjustment for covariates.

3、 Figure 3 presents mixed-effects model analysis of the intervention's effect on Stroop Color-Word Test (SCWT) accuracy. The x-axis represents follow-up time (baseline to post-intervention weeks). In the unadjusted model (Fig. 3A), a significant time × group interaction was observed ($P < 0.01$), with the intervention group showing greater accuracy improvement than controls. After adjusting for covariates (Fig. 3B), this interaction became stronger ($P < 0.01$), indicating faster gains in accuracy within the intervention group. These results confirm that the intervention significantly enhances executive function, measured by SCWT accuracy, with effects strengthened after controlling for confounders, demonstrating independent therapeutic benefits for cognitive interference control.

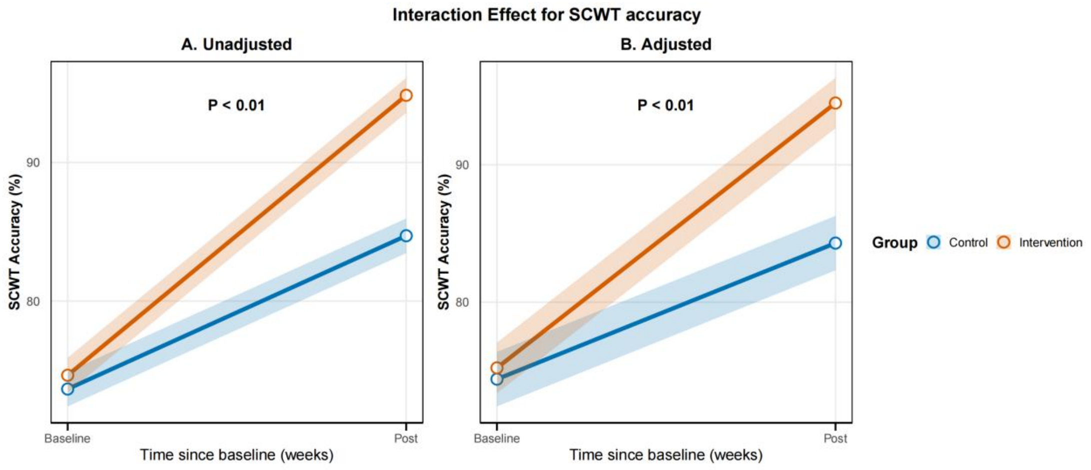


Figure 3 Mixed-effects model analysis of SCWT accuracy. The plots compare the time × group interaction effects before (A) and after (B) adjustment for covariates.

4、 Figure 4 presents mixed-effects model analysis of the intervention's effect on SCWT reaction time. The x-axis represents follow-up time (baseline to post-intervention weeks). In the unadjusted model (Fig. 4A), a significant time × group interaction was observed (P = 0.01), with the intervention group showing greater reduction in reaction time than controls. After adjusting for covariates (Fig. 4B), this interaction became stronger (P < 0.01), indicating faster decreases in reaction time within the intervention group. These results confirm that the intervention significantly improves executive processing speed, reflected by SCWT reaction time, with effects strengthened after controlling for confounders, demonstrating robust independent therapeutic benefits for cognitive interference control.

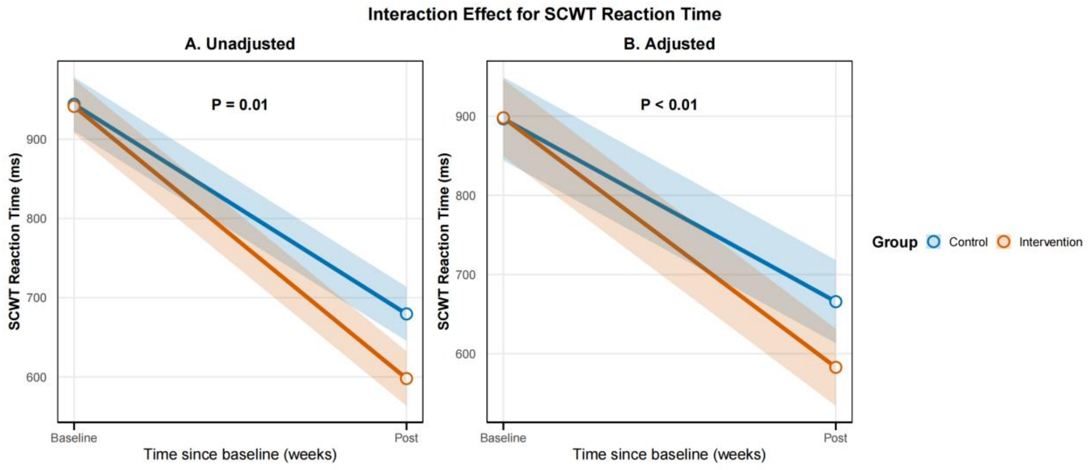


Figure 4 Mixed-effects model analysis of SCWT reaction time. The plots compare the time × group interaction effects before (A) and after (B) adjustment for covariates.

5、 Figure 5 presents mixed-effects model analysis of the intervention's effect on TMT-B accuracy. The x-axis represents follow-up time (baseline to post-intervention weeks). In the unadjusted model (Fig. 5A), a significant time × group interaction was observed (P < 0.01), with the intervention group showing greater accuracy improvement than controls. After adjusting for covariates (Fig. 5B), this interaction remained strong (P < 0.01), indicating faster accuracy gains within the intervention group. These results confirm that the intervention significantly enhances set-shifting and cognitive flexibility, as measured by TMT-B accuracy, with effects maintained after controlling for confounders, demonstrating durable therapeutic benefits for executive function.

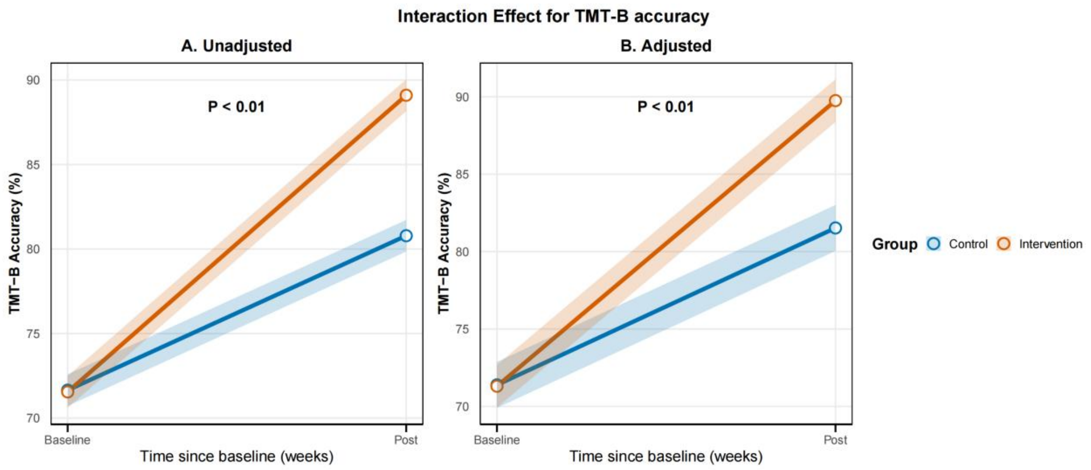


Figure 5 Mixed-effects model analysis of TMT-B accuracy. The plots compare the time × group interaction effects before (A) and after (B) adjustment for covariates.

6、 Figure 6 presents mixed-effects model analysis of the intervention's effect on Trail Making Test Part B (TMT-B) reaction time (seconds). The x-axis represents follow-up time (baseline to post-intervention weeks).In the unadjusted model (Fig. 6A), a significant time × group interaction was observed ($P < 0.01$), with the intervention group showing greater reduction in completion time compared to controls. After adjusting for covariates (Fig. 6B), this interaction remained strong ($P < 0.01$), indicating faster decreases in reaction time within the intervention group. These results confirm that the intervention significantly improves cognitive processing speed and task-switching efficiency, as reflected by reduced TMT-B completion time, with effects maintained after controlling for confounders, demonstrating reliable therapeutic benefits for executive functions related to mental flexibility and visual scanning.

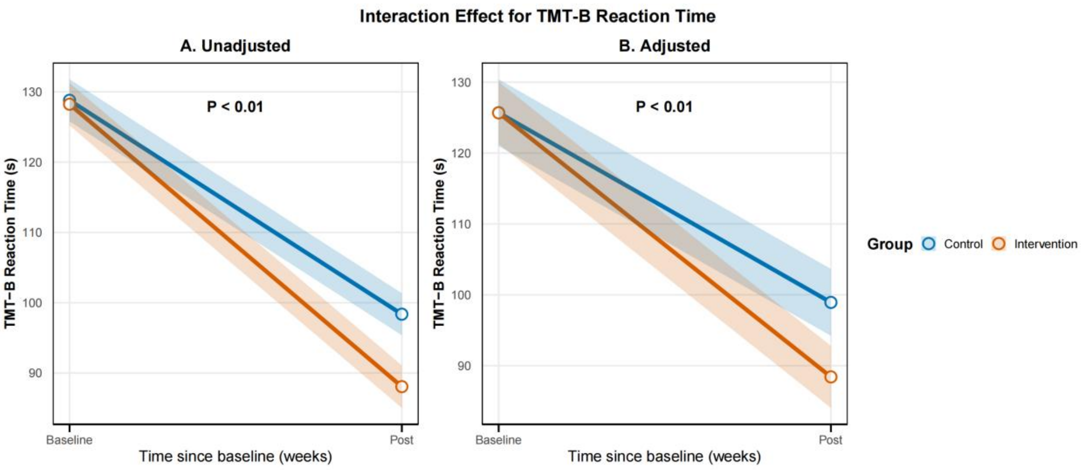


Figure 6 Mixed-effects model analysis of SCWT reaction time. The plots compare the time × group interaction effects before (A) and after (B) adjustment for covariates.

7、 Figure 7 presents mixed-effects model analysis of the intervention's effect on HAMD-17 scores. The x-axis represents follow-up time (baseline to post-intervention weeks). In the unadjusted model (Fig. 7A), a significant time × group interaction was observed ($P < 0.01$), with the intervention group showing greater reduction in depression severity compared to controls. After adjusting for covariates (Fig. 7B), this interaction remained strong ($P < 0.01$), indicating faster symptom improvement in the intervention group. These results confirm that the intervention produces significant antidepressant effects, reflected by decreased HAMD-17 scores, with effects maintained after controlling for confounders, demonstrating robust clinical efficacy in alleviating core depressive symptoms such as mood disturbance, psychomotor retardation, and sleep problems.

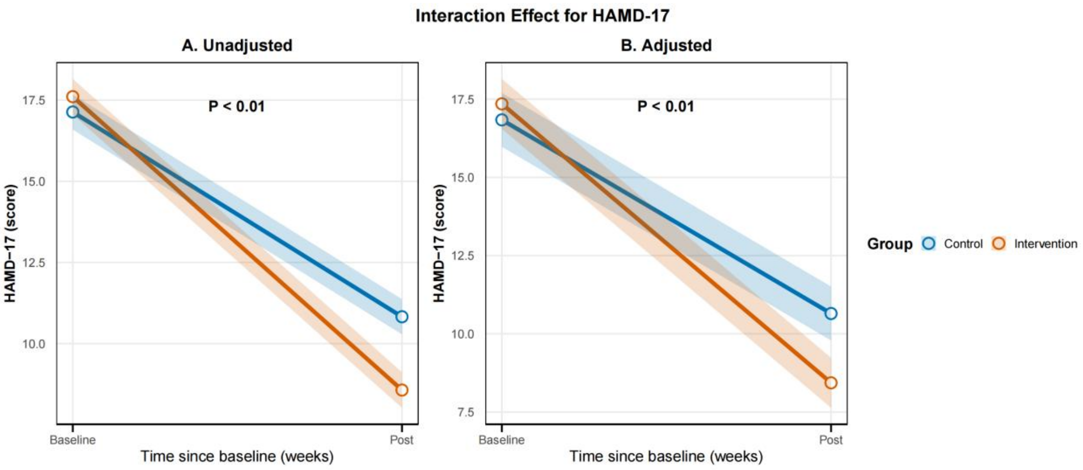


Figure 7 Mixed-effects model analysis of HAMD-17 scores. The plots compare the time ×

group interaction effects before (A) and after (B) adjustment for covariates.

8、 Figure 8 presents mixed-effects model analysis of the intervention's effect on oxy-Hb concentration. The x-axis represents follow-up time (baseline to post-intervention weeks).In the unadjusted model (Fig. 8A), a significant time × group interaction was observed ($P < 0.01$), with the intervention group showing greater increases in oxy-Hb levels compared to controls. After adjusting for covariates (Fig. 8B), this interaction remained strong ($P < 0.01$), indicating faster elevation of oxy-Hb concentration within the intervention group.These results confirm that the intervention significantly enhances prefrontal cortex hemodynamic activity, reflected by increased oxy-Hb, with effects maintained after controlling for confounders, demonstrating improved neurovascular coupling and oxygen metabolism in brain regions involved in cognitive processing.

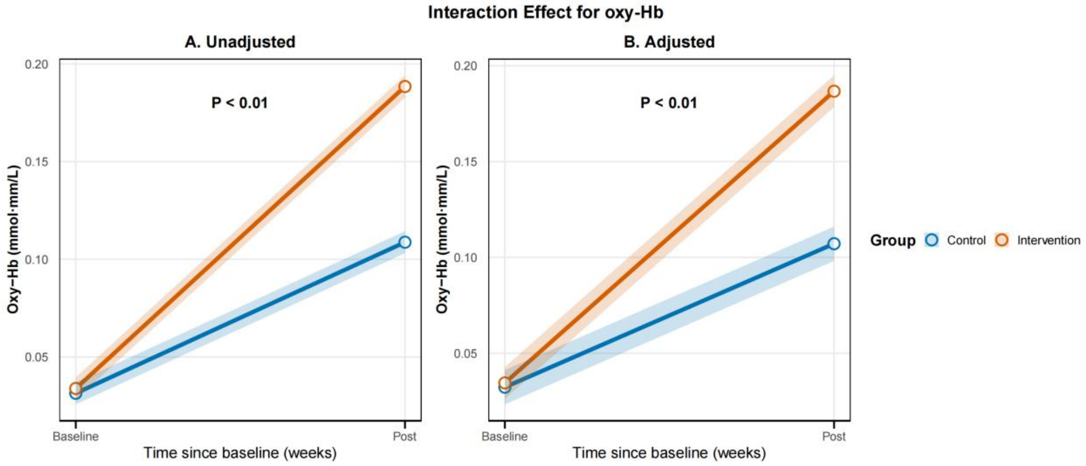


Figure 8 Mixed-effects model analysis of oxy-Hb. The plots compare the time × group interaction effects before (A) and after (B) adjustment for covariates.

9、 Figure 9 presents mixed-effects model analysis of the intervention's effect on neurophysiological integral values. The x-axis represents follow-up time (baseline to post-intervention weeks). In the unadjusted model (Fig. 9A), a significant time × group interaction was observed ($P < 0.01$), with the intervention group showing greater increases in integral values compared to controls. After adjusting for covariates (Fig. 9B), this interaction remained strong ($P < 0.01$), indicating faster elevation of integral measures within the intervention group. These results confirm that the intervention significantly enhances integrated neurovascular or metabolic

activity, reflected by increased integral values, with effects maintained after controlling for confounders, suggesting optimized physiological integration in neural circuits involved in cognitive processing.

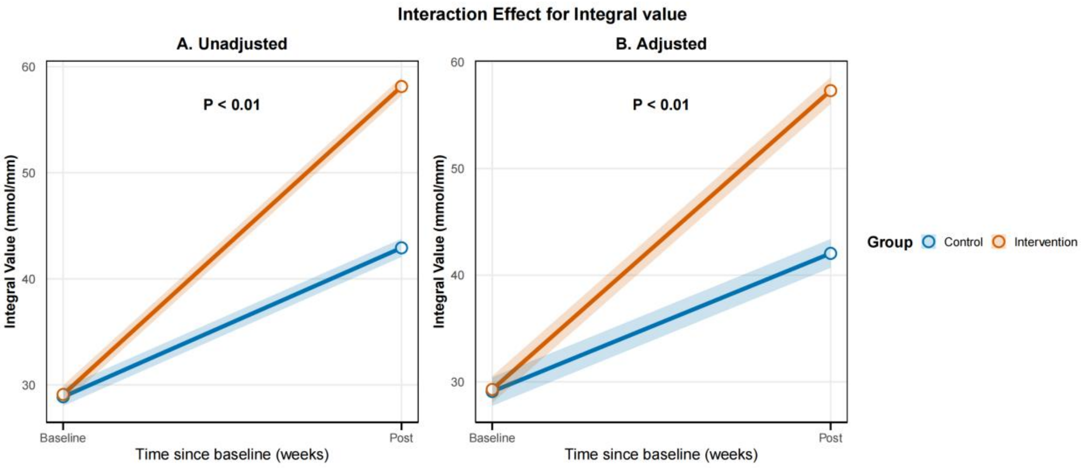


Figure 9 Mixed-effects model analysis of integral values. The plots compare the time × group interaction effects before (A) and after (B) adjustment for covariates.

10、 Figure 10 presents mixed-effects model analysis of the intervention's effect on neurophysiological centroid values. The x-axis represents follow-up time (baseline to post-intervention weeks). In the unadjusted model (Fig. 10A), a significant time × group interaction was observed ($P < 0.01$), with the intervention group showing greater reduction in centroid values compared to controls, indicating more efficient temporal processing. After adjusting for covariates (Fig. 10B), this interaction remained strong ($P < 0.01$), showing faster decreases in centroid measures within the intervention group. These results confirm that the intervention significantly optimizes temporal integration of neurophysiological signals, reflected by decreased centroid values, with effects maintained after controlling for confounders, suggesting enhanced neural processing efficiency and more focused temporal distribution of physiological responses in cognitive networks.

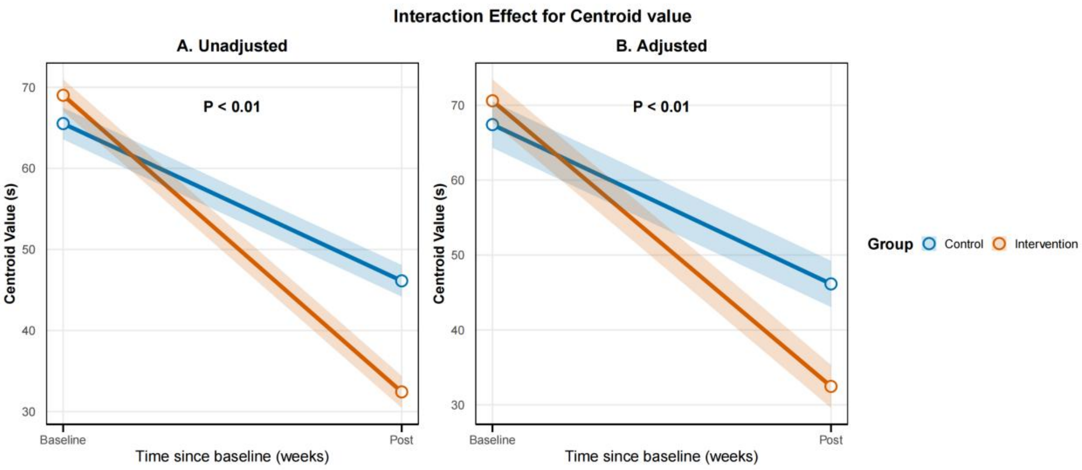


Figure 10 Mixed-effects model analysis of centroid values. The plots compare the time × group interaction effects before (A) and after (B) adjustment for covariates.
